# Factors Influencing Second and Third Dose Observance during Seasonal Malaria Chemoprevention (SMC): A Quantitative Study in Burkina Faso, Mali and Niger

**DOI:** 10.3390/tropicalmed7090214

**Published:** 2022-08-29

**Authors:** Anyirékun Fabrice Somé, Issaka Zongo, Issaka Sagara, Alkassoum Ibrahim, Césaire Damien Ahanhanzo, Edoh Eddie Agbanouvi-agassi, Dona Alain Sayi, Lea Pare Toe, Zachari Kabré, Frédéric Nikiéma, Thomas Bazié, Sylvin Ouédraogo, Issiaka Sombié, Alassane Dicko, Eric Adehossi, Jean-Bosco Ouédraogo, Kounbobr Roch Dabiré

**Affiliations:** 1 Institut de Recherche en Sciences de la Santé, Bobo-Dioulasso BP 545, Burkina Faso; 2 Malaria Research and Training Center (MRTC), Bamako BP 1805, Mali; 3 Faculty of Health Sciences, University Abdou Moumouni of Niamey, Niamey BP 10896, Niger; 4 Organisation Ouest Africaine de la Santé, Bobo-Dioulasso BP 153, Burkina Faso

**Keywords:** seasonal malaria chemoprevention, *Plasmodium falciparum*, Burkina Faso, Mali, Niger

## Abstract

This study aims to evaluate the factors influencing the adherence to the 2nd and 3rd doses of Amodiaquine (AQ) during seasonal malaria chemoprevention (SMC) in Burkina Faso, Mali, and Niger. Overall, 3132 people were interviewed during surveys between 2019 and 2020 in 15 health districts. In Burkina Faso, Mali, and Niger, the proportions of non-adherence were 4.15%, 5.60%, and 13.30%, respectively, for the 2nd dose and 3.98%, 5.60% and 14.39% for the 3rd dose. The main cause of non-adherence to the 2nd and 3rd doses was other illnesses in 28.5% and 29.78%, respectively, in Burkina Faso, 5.35% and 5.35% in Mali and 1.6% and 0.75% in Niger. It was followed by vomiting in 12.24% and 10.63% for Burkina and 2.45% and 3.78% in Niger. The last cause was refusal in 6.12% and 4.25% in Burkina, 33.9% and 15.25% in Mali and 0.8% and 1.51% in Niger. Non-adherence of doses related to parents was primarily due to their absence in 28.5% and 27.65% in Burkina, 16.07% and 16.07% in Mali and 7.37% and 6.06% in Niger. Traveling was the second cause related to parents in 12.24% and 12.76% in Burkina, 19.64% and 19.64% in Mali and 0.81% and 0.75% in Niger. Non-adherence related to community distributors was mainly due to missing the doses in 4.08% and 4.25% in Burkina, 23.21% and 23.21% in Mali, 77.04% and 76.51% in Niger. Our study reported very small proportions of non-adherence to 2nd and 3rd doses of SMC and identified the main causes of non-adherence. These findings will provide helpful information for policymakers and public health authorities to improve adherence to SMC

## 1. Introduction

Despite remarkable efforts deployed to reduce the malaria burden, the disease remains a leading cause of morbidity and mortality in Africa. As such, in 2019 for instance, 229 million cases were reported worldwide and the WHO African Region, with 94% of the cases, is the most affected. Several sub-Saharan countries, including Burkina Faso, Mali, and Niger, are among the major contributors to the global burden of malaria [1]. Moreover, children under 5 years old and pregnant women, who represent 67% of deaths related to malaria, are the most vulnerable populations [1]. Since 2012, seasonal malaria chemoprevention (SMC) has been recommended [2] and is now being implemented in 13 malaria-endemic countries [1]. SMC involves repeated administration of a combination of antimalarial drugs that have both therapeutic and prophylactic effects in areas where transmission is highly seasonal with most cases reported in infants aged 3-59 months [2,3,4]. This strategy targets children under 5 years of age and requires monthly administration of a single curative dose of long-acting sulfadoxine-pyrimethamine (SP) together with three doses of amodiaquine (AQ) [2]. Numerous studies have demonstrated that SMC with AQ-SP is highly effective in reducing malaria cases (uncomplicated and severe), and associated mortality rates [5,6,7,8,9,10,11]. In 2019, SMC was successfully implemented in 13 countries with an average of 21.5 million children under 5 having received AQ-SP monthly [1]. 

Although several SMC implementation strategies have been tested [9,12] in many countries including Burkina Faso, Mali, and Niger; SMC is delivered through a door-to-door approach by community health workers (CHWs). Previous studies have demonstrated a higher coverage when SMC is delivered via such an approach [9,12]. Additionally, the treatment is also available at health facilities and other fixed distribution points [10]. Specifically, in the absence of contraindications, the CHW provides the first dose of AQ with SP to eligible children under direct supervision. After the administration of the first dose, the CHW explains to parents/caregivers how to administer the second and third doses of AQ at home.

Despite the benefit provided by SMC in malaria control strategies, West African countries, including Burkina Faso, Mali and Niger, remain heavily affected by the disease, with high prevalence and mortality rates [13]. A number of studies have reported a high prevalence of asymptomatic malaria or a high incidence of hospital admission or death due to malaria in SMC areas [14,15,16]. The reasons such outcomes persist despite SMC are unknown. Possibilities include failures in (i) the coverage of the monthly course or (ii) lack of observance of the second and/or third doses of AQ by parents or caregivers. Herein, we aimed to assess the factors potentially influencing the adherence to the second and third doses of SMC in Burkina Faso, Mali, and Niger. 

## 2. Materials and Methods

### 2.1. Study Site and Population

The study was conducted in 15 districts across 3 West African countries, namely Burkina Faso, Mali, and Niger. In each country, 5 districts were randomly chosen among those (i) implementing SMC and (ii) receiving funds from the World Bank. In each of the participating districts, surveys were conducted in 10 villages randomly selected from the list of all villages of the district. Our approach yielded a total of 50 villages selected per country (see Supplementary Materials Files S1–S3). In each village, an appropriate sampling based on an approximately constant number of households (refer to Sample size and statistical analysis section) was made from the village list. Surveys were conducted with the approval of the parents or caregivers of children aged 3–59 months. Only one eligible child resident in the household at the time of the survey was included and the parent/caregiver interviewed.

### 2.2. Study Design

This study used a mixed-method research approach involving a quantitative survey coupled with focus group discussions in the same districts that received SMC as part of the World Bank financing campaign (July to October 2018). Both quantitative and qualitative surveys were conducted after SMC campaigns (January to June 2019). Data from the qualitative survey will be published elsewhere. This study exclusively presents the data obtained from the quantitative survey. In each country, five teams each made up of a supervisor, four investigators, and a local guide visited the villages to interview parents/caregivers of eligible children. Each selected village was divided into segments of about 100 households and each segment to be surveyed was randomly chosen. In each selected village, a team of four investigators visited the homes of eligible children after the last SMC cycle to collect relevant information from parents/caregivers. 

### 2.3. Data Collection

Data were collected from parents/caregivers of SMC-eligible children. Briefly, they were asked about their (i) socio-demographic status, (ii) awareness of the SMC program (general knowledge on malaria, SMC coverage per cycle), (iii) number of SMC-eligible children in the household, and (iv) adherence to the second and third SMC doses given at home. Additional information regarding the use of insecticide-treated bed nets and, especially, adverse effects subsequent to administration of SMC were collected. As such, a sufficiently detailed list of possible reasons for not taking the second and third doses was established to anticipate the reasons for non-adherence of any missing dose reported by the parents/caregivers. The reasons not foreseen were listed as “other reasons” (See Supplementary Materials File S4).

### 2.4. Ethical Considerations

The study protocol and the consent form, as well as the data collection materials were approved by the “Comité d’Ethique Institutionnel pour la Recherche en Santé (CEIRES)” of IRSS, the “Comité d’Ethique National pour la Recherche en Santé (CERS)” of Burkina Faso, the “Comité National d’Ethique pour la Santé et les Sciences de la vie” of Mali, and the “Comité National d’Ethique pour la Recherche en Santé” of Niger approved the study. The personal data of participants were kept confidential and were only accessible to study investigators, the ethics committees and the West African Health Organization (WAHO). The participation in the study was completely free and voluntary with the possibility of withdrawing without justification. The informed consent was administered in the participant’s language in the presence of an impartial witness in case the participant was not able to read or write. Children were included only if their parents or caregivers have signed the informed consent. A copy of the signed informed consent was given to each parent/caregiver whose child participated in the project.

### 2.5. Sample Size and Statistical Analysis

Sample sizes were calculated to sufficiently assess the factors that influence observance of the second and third doses of SMC. We assumed that the proportion of children receiving the second and third doses reached 50% in each cluster, i.e., village or community. Considering a non-response rate of 20% and correcting by a factor of 2 to account for the effect of clustering, a power of 80%, and a confidence level of 95% with a margin of error of 0.05; at least 921 children per country or 50 clusters should be included. Data were collected on tablets using an ODK collection platform version1.12 and subsequently analyzed with STATA 16 (Release 16. College Station, TX, USA: StataCorp LLC. StataCorp. 2017.). Chi-square and risk difference were used to compare the different proportions with *p* values < 0.05 considered as statistically significant.

## 3. Results

### 3.1. Baseline Characteristic of the Respondents

In all three countries, the parents/caregivers interviewed were relatively young (suggest adding age data here) and the majority were female (give percent). The highest proportion of men interviewed was noted in Burkina Faso [16.51% (191/1183)]. More than 98% of the study participants were married. Although education levels varied from one country to another, the trends show that illiteracy was high in all three countries, particularly in Burkina Faso, which displayed the highest proportion [65.64% (924/1183), Table 1]. Similarly, the proportion of participants that reached the highest level of education (university) was 1.79% (18/1003) in Mali, 1.06% (10/946) in Niger and only 0.08% (1/1183) in Burkina Faso. Direct parents (mother/father) were most often interviewed during the survey, regardless of the country. A small proportion of grandparents 2% (20/946), 3.38% (40/1183), and 7.5% (71/946) were interviewed in Mali, Burkina Faso, and Niger, respectively (Table 1). 

Regarding knowledge about SMC, at least 96% of respondents were aware of SMC through means such as community relays, healthcare workers, public criers, religious leaders, or local broadcasting radio in Mali and Burkina Faso [96.79% in Burkina Faso and 96.5% in Mali, risk difference = 0.27, 95% CI (−1.24; 1.79), *p* = 0.7191]. On the other hand, the proportion of respondents who heard about the SMC was significantly lower in Niger compared to Burkina Faso [78.96% versus 96.76%, risk difference = −17.82, 95% CI (−20.61; −15.04), *p* < 0.001] and Mali [78.96% versus 96.5%, risk difference = −17.54, 95% CI (−20.38; −14.71), *p* < 0.001]. 

Side effects following the implementation of the SMC were reported in 6.93% (82/1183) of participants in Burkina Faso, 11.57% (116/1003) in Mali, and 20.82% (197/946) in Niger. In Burkina Faso, the most commonly reported side effects were diarrhea/vomiting/weakness (84.15%, 69/82), fever (13.41%, 11/82), or rash and dizziness (1.22%). In Niger, diarrhea/vomiting/weakness represented 81.82% (162/197) of the adverse side effects followed by 9.09% (18/197) for fever, 5.05% for abdominal pain, headache, puffiness of the face, dizziness and pruritis in 3.6% (7/197). In Mali, the trend was sensitively similar as 92.24% (107/116) of the side effects were manifested through diarrhea/vomiting/weakness. Other side effects such as dizziness/lack of appetite (1.72%, 2/116), fever and chills (4.3, 5/116) or unspecified (1.7%, 2/116) were noted.

In Burkina Faso, 95.9% (1135/1183) of participants recognized mosquitoes as the cause of malaria compared to 89.3% (896/1003) in Mali and 92.6% in Niger. When asked about the potential issues subsequent to an absence of treatment, 98.2% (930/947), 96.1% (964/1003), and 98.14% (1161/1183) of the participants in Niger, Mali, and Burkina Faso recognized that malaria is deadly if it is not properly treated. The proportion of participants using bed nets was very high (>90%) in all three countries as seen in Table 1 [90.96% (1076/1183) in Burkina Faso, 95.4% (957/1003) in Mali, and 95.88% (907/946) in Niger] (Table 1).

### 3.2. SMC Coverage and Cards Possession

**Figure 1:**
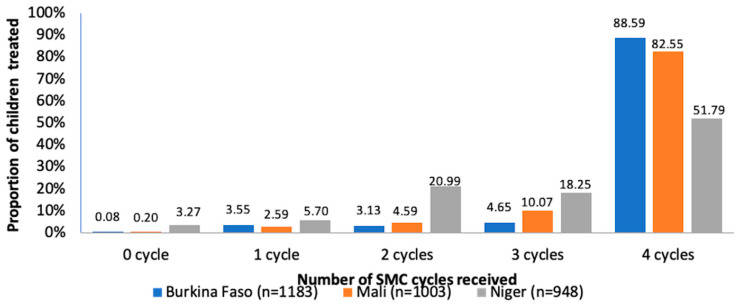
SMC coverage in Burkina Faso, Mali and Niger.

The results show that the proportion of children who did not receive any monthly cycle of SMC was low in general, although it was significantly higher in Niger compared to Burkina Faso and Mali. The cumulative coverage of all four cycles of SMC was significantly higher in Burkina Faso than in Mali [88.59% versus 82.55%, risk difference = 6.04, 95% CI (3.07; −9), *p* < 0.001] and Niger [88.59% versus 51.79%, risk difference = 36.80, 95% CI (33.13; 40.46), *p* < 0.001]. The results indicate that in Niger, the coverage was significantly lower compared to Mali [51.79% versus 82.55%, risk difference = −30.76, 95% CI (−34.71; −26.81), *p* < 0.001]. During the survey, we found that possession of an SMC card was higher in Mali at 88.49% (492/556) compared to Burkina Faso at 76.77% (119/155) and Niger at 83.43% (423/507) (Figure 1).

### 3.3. Non-Adherence of Second and Third Doses of SMC

In Burkina Faso, 4.15% (49/1182) of parents/caregivers did not administer the second dose compared to 3.98% (47/1182) for the third dose (X^2^ = 0.04, *p* = 0.083). In Mali, a similar proportion of parents/caregivers did not proceed with the administration of the second and the third doses [5.60% (56/1001)]. In Niger, 13.30% (122/917) and 14.39% (132/917) of parents/caregivers did not administer the second and third doses, respectively (X^2^ = 0.46, *p* = 0.49), (Table 2). Overall, the proportion of non-adherence for the second dose was comparable between Burkina Faso and Mali [4.15% versus 5.6%, risk difference = −1.45, 95% CI (−3.27; 0.37), *p* = 0.114]. Compared to Niger, these proportions of non-adherence in Burkina and Mali were significantly lower [Burkina Faso vs Niger: 4.15% versus 13.30%, risk difference = −9.16, 95% CI (−11.63; −6.68), *p* < 0.001 and Mali vs Niger: 5.6% versus 13.3%, risk difference = −7.71, 95% CI (−10.33; −5.09), *p* < 0.001]. Non-adherence of the third dose was also significantly lower in Burkina Faso compared to Niger [3.98% and 14.39%, risk difference = −10.42, 95% CI (−12.95; −7.89), *p* < 0.001] and in Mali compared to Niger [5.6% and 14.39%, risk difference = −8.65, 95% CI (−11.31; −5.98), *p* <0.001]. Finally, the proportion of non-adherence of the third dose was similar in Burkina Faso and Mali [3.98% versus 5.6%, risk difference = −1.62, 95%CI (−3.43; 0.19), *p* = 0.0756].

#### 3.3.1. Causes of Non-Adherence of the Second Dose Potentially due to Children

The cause of non-adherence related to children included mainly cases of diseases reported in 28.5% (14/49) in Burkina Faso, 5.35% in Mali, and 1.6% in Niger. It is followed by cases of vomiting following the first dose administration in Burkina Faso [12.24%] and Niger [2.45%]. We also found that some children refused the second dose. They represented 6.12% in Burkina Faso, 33.9% in Mali, and only one case in Niger (Table 3).

#### 3.3.2. Causes of Non-Adherence of the Second Dose due to Parents

The causes of non-adherence to the second dose due to parents not providing the medication were primarily due to the absence of parents during the home visit of the distributor; either temporarily for fieldwork or other activities (28.5% in Burkina Faso, 16.07% in Niger, and 7.37% in Mali) or for traveling out of the village (12.24% in Burkina Faso, 19.64% in Mali and 0.81% in Niger). Furthermore, cases of forgetfulness were noted. One case was reported in Burkina Faso (2.04%) and in Mali (2.45%) while three were found in Niger (Table 3).

#### 3.3.3. Causes of Non-Adherence of the Second Dose due to Community Distributors

The non-delivery of the second dose by community distributors was the main reason reported in Niger (77.04%), Mali (23.21%), and Burkina Faso (4.08%) (Table 3). The differences between the proportion of non-delivery of the second dose noted in these three countries were statistically significant (Niger vs. Mali, X^2^ = 72.81, *p* < 0.001; Niger vs Burkina Faso, X^2^ = 120.26, *p* < 0.001; and Mali vs. Burkina Faso, X^2^ = 9.24, *p* <0.001).

#### 3.3.4. Sociodemographic Characteristics of Parents Who Did Not Administer the Second Dose

The participants who did not administer the second dose were mainly female; 89.8%, 94.65% and 95.9% in Burkina Faso, Mali and Niger, respectively. Parents were married in more than 95% of all three countries. Although their education levels varied in each country, most of the participants who did not administer the second dose were illiterate, and of note, the highest proportion of illiteracy was reported in Burkina Faso [55.10%] followed by Mali [53.57%], and Niger [43.44%]. The proportion of participants with the highest level of education was observed in Niger [2.46%]. Burkina Faso [2.04%] and Mali [1.78%], respectively. 

Respondents who did not administer the second dose had similar awareness of the SMC program in Burkina Faso (89.9%) and Mali (94.65%) [95% CI (−1.24; 1.79), *p* = 0.7191)]. Comparatively, only 65.58% of the participants were aware of the SMC program in Niger. Among those who did not administer the second dose, knowledge of SMC utility was reported in 83.7% of participants in Burkina, 82.14% in Mali, and 76.23% in Niger.

The reported use of bed nets was relatively high in all three countries (>80% as shown in Table 1). In Burkina Faso, 87.75% of those who did not observe the second dose recognized mosquitoes as the cause of malaria vs. 85.71% in Mali and 90.16% in Niger. When asked about potential issues subsequent to non-treated malaria, 99.18%, 98.21%, and 95.92% of those who did not administer the second doses in Niger, Mali, and Burkina Faso recognized that malaria is deadly if not treated properly (Table 4).

#### 3.3.5. Causes of Non-Adherence of the Third Dose Potentially due to Children

The main causes of non-observance of the third dose potentially due to children are represented by sickness of the selected child in 29.78% of cases in Burkina Faso, 5.35% in Mali [risk difference = 0.88, 95% CI (0.18; 1.58), *p* = 0.0191)], and only one case in Niger. We also found 15.25% the children in Mali refused a third dose compared to 4.25% in Burkina Faso [0.9% versus 0.17%, risk difference = 0.73, 95% CI (0.099; 1.36), *p* = 0.06] and 1.51% (2/132) in Niger [0.9% versus 0.2%, risk difference = 0.68, 95% CI (0.02; 0.134), *p* = 0.048] Finally, vomiting was reported in 10.63% in Burkina Faso compared to 8.92% in Niger, and no cases in Mali (Table 3).

#### 3.3.6. Causes of Non-Adherence of the Third Dose Potentially due to Parents

The principal causes of non-observance due to parents were primarily due to temporary absences in 27.65% in Burkina Faso compared to 16.07% in Mali [risk difference = 0.12, 95% CI (−0 75; 0.99), *p* = 0.7917], and 6.06% in Niger [risk difference = 0.22, 95% CI (−0.62; 1.07), *p* = 0 60]. Another cause reported was travel of the parents/child in 19.64 in Mali, 12.76% in Burkina Faso and 0.43% in Niger (Table 3).

#### 3.3.7. Causes of Non-Adherence of the Third Dose Potentially due to Community Distributors

The factors of non-observance of the third dose due to community distributors were largely represented by the non-delivery of the dose to parents, especially in Niger where the proportions of non-delivery of doses reached 76.51% compared to 23.21% in Mali (risk difference 9.72%, 95% CI (7.57–11.86%), *p* < 0.001) and 4.25% in Burkina Faso (risk difference 10.84%, 95% CI 8.81–12.88%, *p* < 0.001) (Table 3). 

#### 3.3.8. Sociodemographic Characteristics of Parents Who Did Not Administer the Third Dose

Among the participants not observing the third doses, 94.64%, 93.18% and 87.23% were female, respectively, in Mali, Niger and Burkina Faso. Those participants were married in more than 95% of all three countries. A large proportion of participants who did not administer the third dose were illiterate with the highest proportion of illiteracy reported in Burkina Faso at 70.20% followed by Mali with 53.57% and Niger at 44.7%. The highest level of education was 3.03% in Niger, 2.13% in Burkina Faso, and 1.78% in Mali. Direct parents (mother/father) were most often interviewed in more than 90% during the survey in all countries.

More than 85% of those who did not administer the third dose were aware of SMC programs in Burkina Faso (87.23%) and Mali (94.64%) 95% CI (−1.24–1.79%), *p* = 0.7191) compared to 65.9% in Niger. Knowledge of SMC utility was 82.98%, 82.14% and 75%, respectively, in Burkina Faso, Mali and Niger. We reported 80.85%, 85.71% and 97.73% of bed-nets use, respectively, in Burkina Faso, Mali and Niger. In Burkina Faso, 87.23% of those who did not administer the third dose recognized mosquitoes as the cause of malaria vs. 83.92% in Mali and 90.9% in Niger. When asked about issues of non-treated malaria, 98.22%, 97.73% and 95.74% of those who did not administer the third doses recognized that malaria is deadly if not properly treated, respectively, in Niger, Mali and Burkina Faso (Table 4).

## 4. Discussion

SMC in children aged 3–59 months remains a promising strategy for malaria control in countries with seasonal malaria transmission. SMC leads to considerable reductions in morbidity and mortality associated with malaria [7,8,17,18,19,20]. Studies in Africa have shown that SMC has a remarkable impact in reducing cases of uncomplicated malaria, hospitalizations and deaths related to malaria [15,18,21,22]. However, despite the benefits of SMC in the fight against malaria, the disease remains a major public health problem in most countries implementing this strategy (WHO, 2018). In controlled clinical trials, SMC drugs were administered under the direct observation of nurses. This approach, although justified may be logistically difficult to implement. In most countries implementing SMC, such as Burkina Faso, Mali, and Niger, only the first dose of SMC is directly observed and the latter two doses are administered by the parents/caregivers, thus raising concerns about the non-adherence to the second and/or third dose compliance. 

Taking into consideration the difficulty to verify the adherence of the second and third doses of SMC which are not supervised by a CHW, this study demonstrated relatively low and comparable proportions of non-adherence to the second doses of SMC reported by parents/caregivers in Burkina Faso and Mali but a significantly higher proportion of non-observance in Niger. Excluding the results from Niger, the results of non-adherence to the second dose reported in this study are comparable to that of a study conducted by Diawara and collaborators in the health district of Kita in Mali [21]. Several factors are associated with non-adherence to the second dose of SMC. Among these factors, there are those potentially due to children which (i) vary from one country to another, (ii) are largely represented by cases of vomiting, (iii) involve illness of children during the SMC cycle, and (iv) show cases of refusal to swallow the drug. The other factors of non-adherence with the second dose potentially due to parents and distributors were travel and the absence of the parents/caregivers of the child, the latter of which represents the majority of the causes of non-adherence in Niger. In Mali, a study reported in 2017 that non-adherence with the second dose was mostly due to parental forgetfulness followed by non-receipt of the second dose [21]. In our study, the causes of non-adherence differed by country. In Burkina Faso, the majority of non-adherence was related to cases of sickness in eligible children, temporary absence of the parents and lastly cases of vomiting of the child. In Mali, non-adherence to the doses is linked to cases of refusal of the child, temporary absences of parents and travel out of the village. Finally, in Niger, the non-adherence to the second dose is mainly linked to the non-receipt of the drug, cases of travel or absence of the parents.

In this study, through reporting of parents/caregivers, we found that a low and comparable proportion of children that did not receive the third dose of SMC in Burkina Faso and Mali (<6%), compared to Niger where more than 14% of children did not receive the third dose. The causes of non-observance of the third dose potentially due to children were mainly cases of sickness, cases of refusal to swallow the medication, and cases of vomiting. The causes of non-adherence to the third dose potentially due to the parents/caregivers and distributors are mainly represented by refusal to swallow the drug in Niger and by absences or travel of the parents/caregivers of the eligible child. It should be noted that a number of parents/caregivers in all three countries failed to provide a reason for not administering the second and third doses of SMC.

The analysis of the socio-demographic characteristics of parents/caregivers who did not administer the second and third doses showed that in the three countries, the majority had knowledge of malaria, its causes, and consequences as well as SMC utility. Most of the parents/caregivers were illiterate married women using long-lasting insecticide-treated bed nets.

Although side effects were recorded, SMC was generally well tolerated in all three countries. The proportion of side effects reported following SMC administration was higher in Niger, compared to Mali and Burkina. These side effects were mostly diarrhea, asthenia/weakness, and vomiting in the three countries.

Despite the challenges related to the distribution of SMC drugs, more than 90% of the children surveyed received at least one cycle of SMC and more than 50% received all four cycles. The cumulative SMC coverage recorded in this study was relatively high in Burkina Faso and Mali with more than 80% of children having received drugs during the four cycles of SMC compared to only 51% in Niger. The proportion of children who did not receive any cycle of SMC was low in Mali and Burkina Faso but slightly above 3% in Niger. This high proportion of coverage reported in Mali and Burkina Faso is comparable to that reported in previous studies [21]. The low cumulative coverage reported in Niger is comparable to that reported by the ACCESS-SMC consortium but significantly lower than the coverage of 83% reported by Salissou and his collaborators in 2016 [23]. The SMC coverage reported in our study demonstrates the importance of outreach and mobilization of parents/caregivers over time to ensure high coverage in all SMC cycles. The high proportion of coverage also corroborates the demonstrated knowledge of parents/caregivers related to the strategy of SMC. Parents are generally in favor of administering the SMC drugs. This strong support and favorable opinion towards the implementation of SMC are major assets for the continuity of SMC and are also reflected in the high rates of administration/observance of the second- and third-day treatments by parents at home.

One of the limitations of this study is that it was not conducted immediately after the last cycle of SMC in 2018 for logistical reasons. Moreover, the results obtained from this quantitative survey related to adherence were not compared with what was written on the SMC cards available to children. However, to the best of our knowledge, since the large-scale implementation of SMC, this study is among a limited number that have assessed adherence to second and third doses of SMC drugs in three different countries implementing the SMC strategy.

## 5. Conclusions

This study reported a relatively low proportion of non-adherence to the second and third doses of SMC drugs in Burkina Faso and Mali and a higher proportion in Niger. Non-adherence to the SMC drugs is mainly due to children being ill during the distributor visits, absence of parents/caregivers and drugs not being received by parents/caregivers. Our findings will provide helpful information for policymakers and public health authorities to improve adherence in SMC-implementing countries.

## Figures and Tables

**Table 1:** Baseline characteristics of the survey’s respondents.

	Burkina Faso	Mali	Niger
Proportion of total sample size% (*n*/N)	38 (1183/3132)	32 (1003/3132)	30 (946/3132)
Age (years)			
Mean (SD)	31.10 (9.44)	30/30.45 (8.54)	30/31.68 (10.35)
Minimum-Maximum	15–75	15–75	15–70
Sex			
Male *n* (%)	191 (16.15)	56 (5.58)	63 (6.60)
Female *n* (%)	992 (83.85)	947 (94.42)	883 (93.40)
Marital status			
Married *n* (%)	1164 (98.39)	1000 (99.7)	933 (98.5)
Single *n* (%)	19 (1.61)	3 (0.3)	14 (1.5)
Education level			
Illiterate *n* (%)	924 (65.64)	551 (54.94)	362 (38.27)
Middle school *n* (%)	31 (2.62)	40 (3.99)	148 (15.64)
Primary school *n* (%)	109 (9.21)	203 (20.24)	181 (19.13)
Koranic school *n* (%)	75 (6.34)	84 (8.37)	212 (22.41)
High school *n* (%)	21 (1.78)	40 (4)	16 (1.69)
Literate *n* (%)	112 (9.47)	42 (4.19)	12 (1.27)
University *n* (%)	1 (0.08)	18 (1.79)	10 (1.06)
Others (pre-school, etc)	6 (0.5)	14 (1.40)	5(0.53)
Relation of the respondent with the child			
Father/mother *n* (%)	1117 (94.42)	971 (96.81)	857 (90.59)
Grandparents *n* (%)	40 (3.38)	20 (2)	71 (7.5)
Others (aunt, brother, sister, uncle) *n* (%)	26 (2.20)	12 (1.9)	17 (1.80)
Knowledge of SMC			
Yes *n* (%)	1145 (96.79)	968 (96.5)	747 (78.96)
No *n* (%)	38 (3.21)	35 (3.5)	199 (21.04)
Knowledge of SMC utility			
Yes *n* (%)	1096 (92.65)	876 (87.34)	811 (85.73)
No *n* (%)	82 (6.93)	113 (11.27)	68 (7.20)
Do not know *n* (%)	5 (0.42)	14 (1.4)	67 (7.08)
Use of bednets *n* (%)			
Yes *n* (%)	1076 (90.96)	957 (95.41)	907 (95.88)
No *n* (%)	107 (9.04)	46 (4.59)	39 (4.12)
Knowledge of adverse events of SMC			
Yes *n* (%)	82 (6.93)	116 (11.57)	197 (20.82)
No *n* (%)	1101 (93.07)	887 (88.43)	749 (79.17)
Do not know *n* (%)	0	0	16 (1.59)
Knowledge of malaria causes			
Yes (mosquitoes) *n* (%)	1135 (95.94)	896 (89.33)	876 (92.6)
Inadequate (any other responses) *n* (%)	43 (3.63)	92 (9.17)	54 (5.71)
Do not know *n* (%)	3 (0.25)	11 (1.1)	4 (0.4)
Knowledge of death as consequences			
Yes *n* (%)	1161 (98.14)	964 (96.11)	930 (98.31)
No *n* (%)	16 (1.35)	29 (2.89)	6 (0.6)
Do not know *n* (%)	6 (0.51)	10 (1)	10 (1.06)

**Table 2:** Frequencies of non-adherence of doses 2 and 3 in Burkina Faso, Mali and Niger.

Country	Administration of Dose 2	Administration of Dose 3		Total
		Yes	No	
Burkina Faso	Yes	1131	2	1133
	No	4	45	49
Mali	Yes	945	0	945
	No	0	56	56
Niger	Yes	782	13	795
	No	3	119	122
Total		2865	235	3100

**Table 3:** Causes of non-adherence of the second and third doses in Burkina Faso, Mali and Niger.

	Burkina Faso		Mali		Niger	
Potentially Related to Children	2nd Dose (N = 49)	3rd Dose (N = 47)	2nd Dose (N = 56)	3rd Dose (N = 56)	2nd Dose (N = 122)	3rd Dose (N = 132)
Disease other than malaria *n* (%)	14 (28.5)	14 (29.78)	3 (5.35)	3 (5.35)	2 (1.6)	1 (0.75)
Vomiting *n* (%)	6 (12.24)	5 (10.63)	0	0	3 (2.45)	5 (3.78)
Refusal *n* (%)	3 (6.12)	2 (4.25)	19 (33.9)	9 (15.25)	1(0.8)	2 (1.51)
Potentially related to parents						
Absence of parents *n* (%)	14 (28.5)	13 (27.65)	9 (16.07)	9 (16.07)	9 (7.37)	8 (6.06)
Travel *n* (%)	6 (12.24)	6 (12.76)	11 ((19.64)	11 (19.64)	1 (0.81)	1 (0.75)
Forgetting *n* (%)	1 (2.04)	2 (4.25)	1 (1.78)	1 (1.78)	3(2.45)	4 (3.03)
Child treated with antimalarial *n* (%)	1 (2.04)	0	0	0	0	1(0.75)
No trust in SMC drugs *n* (%)	0	0	0	8 (14.28)	1 (0.81)	0
Waiting for next disease episode *n* (%)	0	0	1 (1.78)	1 (1.78)	0	0
Problem with previous cycle *n* (%)	0	1	0	0	1 (0.81)	1 (0.75)
SMC card (lost, not received…) etc.) *n* (%)	0	0	8 (14.28)	0	0	1 (0.75)
Potentially related community distributors						
Missing doses *n* (%)	2 (4.08)	2 (4.25)	13 (23.21)	13 (23.21)	94 (77.04)	101 (76.51)
Drugs out of stock *n* (%)	2 (4.08)	2 (4.25)	0	0	0	0
Not informed *n* (%)	0	0	1 (1.78)	1 (1.78)	0	0
Drug administration not explained *n* (%)	0	0	0	0	1 (0.81)	1 (0.75)
No Answer or no reason *n* (%)	0	0	0	0	5 (4.10)	1 (0.75)
Missing data *n* (%)	0	0	0	0	1 (0.81)	5 (3.78)
Total *n* (%)	49 (100)	47(100)	56 (100)	56 (100)	122 (100)	132 (100)

N: number of respondents who did not administer the dose; *n*: number of causes of non-adherence.

**Table 4:** Sociodemographic characteristics of parents who did not administer the second and third doses.

	Burkina Faso		Mali		Niger	
Sex of Respondents	2nd Dose (N = 49)	3rd Dose (N = 47)	2nd Dose (N = 56)	3rd Dose (N = 56)	2nd Dose (N = 122)	3rd Dose (N = 132)
Female *n* (%)	44 (89.8)	41 (87.23)	53 (94.65)	53 (94.64)	117 (95.90)	123 (93.18)
Male *n* (%)	5 (10.20)	6 (12.77)	3 (5.35)	3 (5.36)	5 (4.10)	9 (6.82)
Marital status of respondents						
Married *n* (%)	47 (95.92)	45 (95.74)	56 (100)	56 (100)	119 (97.54)	129 (97.73)
Single *n* (%)	2 (4.08)	2 (4.26)	0	0	3 (2.46)	3 (2.27)
Education level of respondents						
Illiterate *n* (%)	27 (55.10)	33 (70.20)	30 (53.57)	30 (53.57)	53 (43.44)	59 (44.70)
Middle school *n* (%)	1 (2.04)	1 (2.13)	0	1 (1.78)	24 (19.67)	27 (20.45)
Primary school *n* (%)	3 (6.12)	3 (6.38)	18 (32.14)	18 (32.14)	24 (19.67)	25 (18.94)
Koranic school *n* (%)	1 (2.04)	2 (4.26)	4 (7.14)	3 (5.36)	17 (13.93)	15 (11.36)
High school *n* (%)	1 (2.04)	1 (2.13)	1 (1.78)	1 (1.78)	3 (2.46)	4 (3.03)
Literate *n* (%)	5 (10.20)	6 (12.77)	1 (1.78)	1 (1.78)	0	0
University *n* (%)	0	1 (2.13)	2 (3.57)	2 (3.57)	1 (0.82)	2 (1.51)
Relationship of respondents with the child						
Father/mother *n* (%)	45 (91.83)	43 (91.49)	55 (98.21)	55 (98.22)	117 (95.90)	127 (96.21)
Grand-parents *n* (%)	1 (2.04)	1 (2.13)	0	0	4 (3.28)	4 (3.03)
Other (aunts, brothers …) *n* (%)	3 (6.12)	3 (6.38)	1 (1.78)	1 (1.78)	1 (0.82)	1 (0.76)
Aware of SMC program						
Yes *n* (%)	44 (89.8)	41 (87.23)	53 (94.65)	53 (94.64)	80 (65.58)	87 (65.91)
No *n* (%)	5 (10.20)	5 (12.77)	3 (5.35)	3 (5.36)	42 (34.42)	45 (34.09)
knowledge of SMC utility						
Protect against malaria *n* (%)	41 (83.67)	39 (82.98)	46 (82.14)	46 (82.14)	93 (76.23)	99 (75)
Do not know SMC utility *n* (%)	8 (16.33)	8 (17.02)	10 (17.86)	6 (10.72)	16 (13.11)	17 (12.88)
No response *n* (%)	0	0	0	4 (7.14)	13 (10.66)	16 (12.12)
bed-nets use						
Yes *n* (%)	40 (81.63)	38 (80.85)	48 (85.71)	48 (85.71)	119 (97.54)	129 (97.73)
No *n* (%)	9 (18.37)	9 (19.15)	8 (14.29)	8 (14.29)	3 (2.46)	3 (2.27)
Presence of adverse events after SMC						
Yes *n* (%)	10 (20.41)	11 (22.44)	0	5 (8.93)	18 (14.75)	22 (16.67)
No *n* (%)	39 (79.59)	36 (3.04)	56 (100)	51 (91.07)	104 (85.25)	110 (83.33)
Knowledge of malaria cause						
Yes (mosquitoes) *n* (%)	43 (87.75)	41 (87.23)	48 (85.71)	47 (83.92)	110 (90.16)	120 (90.90)
Any response other than mosquitoes *n* (%)	6 (12.25)	6 (12.77)	6 (10.72)	7 (12.50)	11 (9.02)	11 (18.34)
Do not know *n* (%)	0	0	2 (3.57)	2 (3.58)	1 (0.82)	1 (0.76)
Knowledge of malaria consequences						
Yes *n* (%)	47 (95.92)	45 (95.74)	55 (98.21)	55 (98.22)	121 (99.18)	129 (97.73)
No *n* (%)	2 (4.08)	2(4.26)	1 (1.79)	1 (1.78)	1 (0.82)	1 (0.76)
Do not know *n* (%)	0	0	0	0	0	2 (1.51)

N: number of respondents who did not administer the dose; *n*: number of cases.

## Data Availability

All data supporting the reported results are available on request.

## Supplementary Materials

The following supporting information can be downloaded at: https://www.mdpi.com/article/10.3390/tropicalmed7090214/s1, File S1: Geographic representation of the study area in Burkina Faso; File S2: Geographic representation of the study area in Mali; File S3: Geographic representation of the study area in Niger; File S4: Quantitative data collection form.

## References

[1] World Health Organization (2020). World Malara Report 2020: 20 Years of Global Progress and Challenges.

[2] World Health Organization (2012). WHO Policy Recommendation: Seasonal Malaria Chemoprevention (SMC) for Plasmodium falciparum Malaria Control in Highly Seasonal Transmission Areas of the Sahel Sub-Region in Africa.

[3] Greenwood B. (2006). Review: Intermittent preventive treatment—A new approach to the prevention of malaria in children in areas with seasonal malaria transmission. Trop. Med. Int. Health.

[4] Cairns M., Roca-Feltrer A., Garske T., Wilson A.L., Diallo D., Milligan P.J., Ghani A.C., Greenwood B.M. (2012). Estimating the potential public health impact of seasonal malaria chemoprevention in African children. Nat. Commun..

[5] Cisse B., Sokhna C., Boulanger D., Milet J., Ba E.H., Richardson K., Hallett R., Sutherland C., Simondon K., Simondon F. (2006). Seasonal intermittent preventive treatment with artesunate and sulfadoxine-pyrimethamine for prevention of malaria in Senegalese children: A randomised, placebo-controlled, double-blind trial. Lancet.

[6] Kweku M., Liu D., Adjuik M., Binka F., Seidu M., Greenwood B., Chandramohan D. (2008). Seasonal intermittent preventive treatment for the prevention of anaemia and malaria in Ghanaian children: A randomized, placebo controlled trial. PLoS ONE.

[7] Dicko A., Diallo A.I., Tembine I., Dicko Y., Dara N., Sidibe Y., Santara G., Diawara H., Conare T., Djimde A. (2011). Intermittent preventive treatment of malaria provides substantial protection against malaria in children already protected by an insecticide-treated bednet in Mali: A randomised, double-blind, placebo-controlled trial. PLoS Med..

[8] Konate A.T., Yaro J.B., Ouedraogo A.Z., Diarra A., Gansane A., Soulama I., Kangoye D.T., Kabore Y., Ouedraogo E., Ouedraogo A. (2011). Intermittent preventive treatment of malaria provides substantial protection against malaria in children already protected by an insecticide-treated bednet in Burkina Faso: A randomised, double-blind, placebo-controlled trial. PLoS Med..

[9] Bojang K.A., Akor F., Conteh L., Webb E., Bittaye O., Conway D.J., Jasseh M., Wiseman V., Milligan P.J., Greenwood B. (2011). Two strategies for the delivery of IPTc in an area of seasonal malaria transmission in the Gambia: A randomised controlled trial. PLoS Med..

[10] (2020). ACCESS-SMC Partnership. Effectiveness of seasonal malaria chemoprevention at scale in west and central Africa: An observational study. Lancet.

[11] Kirakoya-Samadoulougou F., De Brouwere V., Fokam A.F., Ouédraogo M., Yé Y. (2022). Assessing the effect of seasonal malaria chemoprevention on malaria burden among children under 5 years in Burkina Faso. Malar. J..

[12] Barry A., Issiaka D., Traore T., Mahamar A., Diarra B., Sagara I., Kone D., Doumbo O.K., Duffy P., Fried M. (2018). Optimal mode for delivery of seasonal malaria chemoprevention in Ouelessebougou, Mali: A cluster randomized trial. PLoS ONE.

[13] World Health Organisation (2018). High Burden to High Impact: A Target Malaria Response.

[14] Cairns M., Ceesay S.J., Sagara I., Zongo I., Kessely H., Gamougam K., Diallo A., Ogboi J.S., Moroso D., Van Hulle S. (2021). Effectiveness of seasonal malaria chemoprevention (SMC) treatments when SMC is implemented at scale: Case-control studies in 5 countries. PLoS Med..

[15] Cairns M.E., Sagara I., Zongo I., Kuepfer I., Thera I., Nikiema F., Diarra M., Yerbanga S.R., Barry A., Tapily A. (2020). Evaluation of seasonal malaria chemoprevention in two areas of intense seasonal malaria transmission: Secondary analysis of a household-randomised, placebo-controlled trial in Houndé District, Burkina Faso and Bougouni District, Mali. PLoS Med..

[16] Coldiron M.E., Assao B., Guindo O., Sayinzoga-Makombe N., Koscalova A., Sterk E., Quere M., Ciglenecki I., Mumina A., Atti S. (2021). Prevalence of malaria in an area receiving seasonal malaria chemoprevention in Niger. Malar. J..

[17] Wilson N.O., Ceesay F.K., Obed S.A., Adjei A.A., Gyasi R.K., Rodney P., Ndjakani Y., Anderson W.A., Lucchi N.W., Stiles J.K. (2011). Intermittent preventive treatment with sulfadoxine-pyrimethamine against malaria and anemia in pregnant women. Am. J. Trop. Med. Hyg..

[18] Wilson A.L. (2011). A systematic review and meta-analysis of the efficacy and safety of intermittent preventive treatment of malaria in children (IPTc). PLoS ONE.

[19] Maiga H., Barger B., Sagara I., Guindo A., Traore O.B., Tekete M., Dara A., Traore Z.I., Diarra M., Coumare S. (2020). Impact of Three-Year Intermittent Preventive Treatment Using Artemisinin-Based Combination Therapies on Malaria Morbidity in Malian Schoolchildren. Trop. Med. Infect. Dis..

[20] Maiga H., Gaudart J., Sagara I., Diarra M., Bamadio A., Djimde M., Coumare S., Sangare B., Dicko Y., Tembely A. (2020). Two-Year Scale-Up of Seasonal Malaria Chemoprevention Reduced Malaria Morbidity among Children in the Health District of Koutiala, Mali. Int. J. Environ. Res. Public Health.

[21] Diawara F., Steinhardt L.C., Mahamar A., Traore T., Kone D.T., Diawara H., Kamate B., Kone D., Diallo M., Sadou A. (2017). Measuring the impact of seasonal malaria chemoprevention as part of routine malaria control in Kita, Mali. Malar. J..

[22] Druetz T., Corneau-Tremblay N., Millogo T., Kouanda S., Ly A., Bicaba A., Haddad S. (2018). Impact Evaluation of Seasonal Malaria Chemoprevention under Routine Program Implementation: A Quasi-Experimental Study in Burkina Faso. Am. J. Trop. Med. Hyg..

[23] Issa S., Lamine M.M., Yerima B., Ibrahim A., Djakou H., Maman L.I. (2016). Perception de la chimioprévention du paludisme saisonnier au Niger. Int. J. Biol. Chem. Sci..

