# Supplementary material for: Factors Influencing Second and Third Dose Observance during Seasonal Malaria Chemoprevention (SMC): A Quantitative Study in Burkina Faso, Mali and Niger
Source: Trop Med Infect Dis. 2022 Aug 29;7(9):214. doi: 10.3390/tropicalmed7090214 (PMC9503675; doi:10.3390/tropicalmed7090214)
Supplement: Supplementary file 1 [file tropicalmed-07-00214-s001.zip › S2-Geographic representation of study area in Mali.pdf]

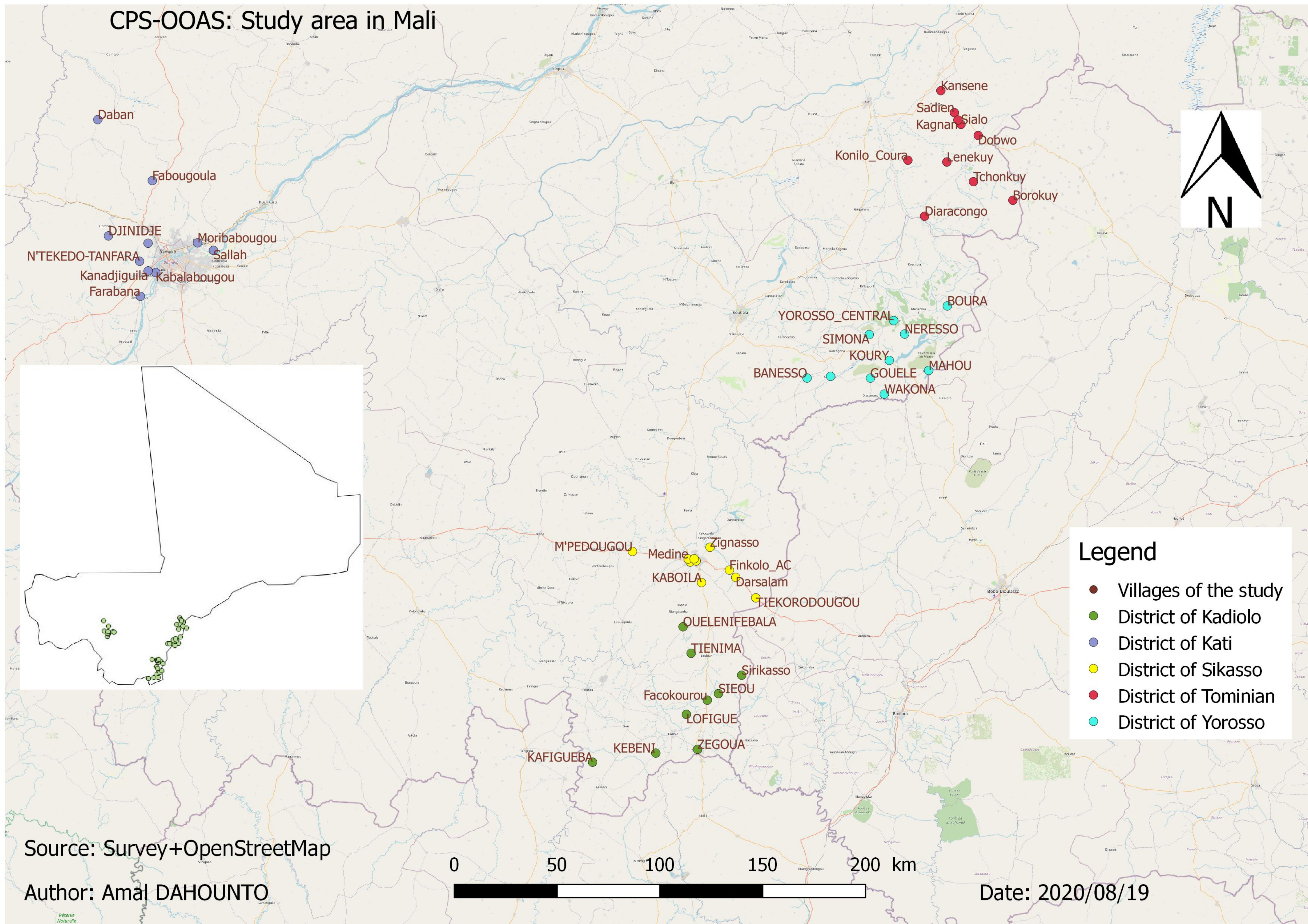
CPS-OOAS: Study area in Mali
Daban
Fabougoula
DJINIDJE
Moribabougou
N'TEKEDO-TANFARA
Sallah
Kanadjiguila
Kabalabougou
Farabana
Kansene
Sadien
Sialo
Kagnan
Dobwo
Konilo_Coura
Lenekuy
Tchonkuy
Borokuy
Diaracongo
BOURA
YOROSSO_CENTRAL
NERESSO
SIMONA
KOURY
BANESSO
GOUELE
MAHOU
WAKONA
M'PEDOUGOU
Zignasso
Medine
Finkolo_AC
KABOILA
Darsalam
TIEKORODOUGOU
OUELENIFEBALA
TIENIMA
Sirikasso
SIEOU
Facokourou
LOFIGUE
KAFIGUEBA
KEBENI
ZEGOUA
N
Legend
Villages of the study
District of Kadiolo
District of Kati
District of Sikasso
District of Tominian
District of Yorosso
0 50 100 150 200 km
Source: Survey+OpenStreetMap
Author: Amal DAHOUNTO
Date: 2020/08/19
