# Supplementary material for: Factors Influencing Second and Third Dose Observance during Seasonal Malaria Chemoprevention (SMC): A Quantitative Study in Burkina Faso, Mali and Niger
Source: Trop Med Infect Dis. 2022 Aug 29;7(9):214. doi: 10.3390/tropicalmed7090214 (PMC9503675; doi:10.3390/tropicalmed7090214)
Supplement: Supplementary file 1 [file tropicalmed-07-00214-s001.zip › S4_Quantitative data collection form.pdf]

Annexe A : Fiche de collecte des informations

Enquête sur les facteurs influençant l'observance des 2em et 3em dose de CPS

Renseignements sur les enfants

Prénom et nom de l'enfant : **Date de naissance ou âge de l'enfant :** **Sexe : 1❑ M 2❑ F**

Numéro ID de l'enfant : /______________/ **Prénom et nom de la mère :**

Tel :

Nom de l'enquêteur : **Date de la visite : /___/_____/______/** **Village :**

District : **CSPS :** **_Village/secteur ______________________________**

Nombre d'enfants dans le ménage

Combien d'enfants de 3 mois à 10 ans vivent dans cette concession ?
Combien de ces enfants avait entre 3 et 59 mois ET ont reçu la CPS lors de la visite des relais communautaires / distributeurs ? (rappel)
Combien de ces enfants avait entre 5 et 10 ans ET ont reçu la CPS lors de la visite des relais communautaires / distributeurs (rappel)
Demandez de contrôler la carte CPS : combien de cartes CPS vous observez (enfants traités, par carte)
Combien de ces cartes sont pour des enfants entre 3 et 59 mois ?
Combien de ces cartes sont pour des enfants entre 5 et 10 ans ?

Caractéristiques de l'enfant sélectionné pour l'enquête CPS

L'enfant est-il présent ? 1❑ Oui 2❑ Non Si Non, Motif absence ……………………………… Lieu ………………………………………

Nb d'années dans le village ? |___| Années Ou de mois |___| Mois

L'enfant est-il résident ? 1❑ Oui 2❑ Non (vécu 6 mois ou intention de rester pendant au moins six mois)

Nom et Prénom de celle (ou celui) qui s'occupe de l'enfant ______________________________ Sexe |___| M/F Âge |___|___| années

1=Mère, 2=Père, 3=Sœur, 4=Grand-mère, 5=Tante, 6=Autre:

Quelle est la relation avec l'enfant ? |___|

Statut matrimonial : 1❑ Marié(e) 2❑ Célibataire 3❑ Veuve 4❑ Divorcée

Cette personne a t elle été à l'école coranique ? 1❑ Oui 2❑ Non si oui, combien d'années d'école au total? |____|____| Années

Cette personne a t-elle été à l'école française? 1❑ Oui 2❑ Non

si oui, combien d'années d'école au total? |___|___| Années ou Niveau d'étude /_____________/

Connaissances sur le paludisme

Comment attrape-t-on le paludisme ? (Plusieurs réponses possibles) 1❑ Le vent 2❑ La pluie 3❑ Les moustiques 4 ❑ les saletés 5❑ Les esprits 9❑ Autres à préciser : …………….

Quels sont les signes du paludisme ? (Plusieurs réponses possibles) 1 ❑ Les maux de tête 2 ❑ Les frissons et les sueurs 3❑ Les Vomissements 4❑ La toux 5 ❑ Le corps chaud

9❑ Autres à préciser : ……………………………………….

Peut – on mourir du paludisme ? 1❑ Oui 2❑ Non 3 ❑ Nsp

Votre enfant a-t-il eu le paludisme ces deux dernières semaines ? |___| Oui /Non /NSP si oui, a-t-il pris un traitement|___| Oui /Non /NSP

si oui, où l'a-t-il pris 1, 2, 3, 4, 5, 6, 7, 8,9,

1 : à la maison 2 : case de santé 3 : poste de santé 4 : centre de santé 5 : hôpital 6 : boutique 7 : pharmacie 8 : marché hebdomadaire 9 : guérisseur

Renseignements sur l'utilisation des moustiquaires

L'enfant dort-il d'habitude sous une moustiquaire ? |___| Oui /Non/NSP, quelle est la dernière fois qu'il a dormi sous moustiquaire : jours |___| semaines |___| Mois |___| JAMAIS |___|

Pendant la saison des pluies dort-il sous moustiquaire : Toutes les nuits |___| la plupart des nuits |___| quelques nuits |___| jamais |___|

L'enfant a-t-il dormi sous moustiquaire la nuit dernière ? |___| Oui /Non/Nsp

D'où vient la moustiquaire ? Achetée |___| Etat |___| ONG |___| Si oui lequel ________

Cette moustiquaire a-t-elle été traitée d'insecticide au moins 1 fois lors les 12 derniers mois? |___| Oui/ Non/ Nsp

Si Non : Moustiquaire de n'importe quel type ? |___| Oui /Non/Nsp Moustiquaire imprégné ? |___| Oui /Non/Nsp Milda ? |___| Oui /Non/Nsp

Cette moustiquaire Est-elle intacte (<5 trous) ? |__| Oui/Non

Renseignements sur la CPS:

Avez-vous entendu parler de la CPS? 1❑ Oui 2❑ Non

Si Oui, à quelle occasion ? 1❑ Au centre de santé 2❑ A la radio 3❑ A la télévision 4. Par votre voisin 5-Agent de santé communautaire, 6- Leaders locaux ; 7 Leaders religieux (église/mosquée) ; 8 Affiches et dépliants ; 9❑ Autres à préciser : ………………………..

A quoi sert-elle ?   1❑ Protège contre le paludisme   2❑ Protège contre les moustiques   3❑ Baisse la fièvre   4❑ Je ne sais pas   9❑Autres à préciser :………………………………….

Les médicaments de CPS, peuvent-ils donner des réactions secondaires aux enfants? 1❑ Oui 2❑ Non

L'enfant a-t-il reçu au moins un cycle de CPS ? 1❑ Oui   2❑ Non   Si Non motif : /____________________/

L'enfant a-t-il reçu les 4 cycles de CPS? 1❑ Oui   2❑ Non   Si Non motif : /____________________/

A	**Si non, pourquoi ?**<br>1 Responsable de l'enfant et enfant absents lors de la visite<br>2 Mari absent donc impossible d'avoir sa permission<br>3 Refus d'administrer le médicament à l'enfant<br>4 Ménage non visité par les agents de santé communautaires<br>5 L'enfant était malade<br>6 L'enfant est allergique aux médicaments<br>7 Toute autre réponse
B	**Si refus, pourquoi ?**<br>1 Mari absent donc impossible d'avoir sa permission<br>2 Le partenaire a refusé<br>3 Médicaments dangereux<br>4 Raison religieuse<br>5 Toute autres réponses

Si non, cocher le cycle non reçu par l'enfant 1❑ 2❑ 3❑ 4❑

L'enfant était-il présent lors de l'administration de CPS au mois de Oct/Nov? 1❑ Oui   2❑ Non

Si Non motif : /____________________/

A- t-il été nécessaire de recueillir l'aval d'une tierce personne avant administration de CPS à l'enfant ?

1❑ Oui   2❑ Non

Si Oui, qui est cette personne ? 1❑ Mère 2❑ Père 3❑ Sœur 4❑ Grand-mère 5❑ Tante 6❑

Autre à préciser :………………..…

L'enfant a-t-il la Carte CPS ? 1❑ Oui   2❑ Non   Si Non, une carte CPS lui a-t-elle été délivrée ?

1❑ Oui   2❑ Non   3❑ Nsp

Mentionnez (et vérifier aussi sur la carte) si les traitements CPS ont été reçus chaque mois et si cela n'a pas été le cas, demandez la raison

Mois	Traitement
**Juliet**	1❑ Oui 2❑ Non 3❑ Nsp si non, précisez la raison |__| 1, 2, 3, 4, 5, 6, 7, 8

**Août**	1❑ Oui 2❑ Non 3❑ Nsp	si non, précisez la raison |__| 1, 2, 3, 4, 5, 6, 7, 8
**Septembre**	1❑ Oui 2❑ Non 3❑ Nsp	si non, précisez la raison |__| 1, 2, 3, 4, 5, 6, 7, 8
**Octobre**	1❑ Oui 2❑ Non 3❑ Nsp	si non, précisez la raison |__| 1, 2, 3, 4, 5, 6, 7, 8
**Novembre**	1❑ Oui 2❑ Non 3❑ Nsp	si non, précisez la raison |__| 1, 2, 3, 4, 5, 6, 7, 8

1=enfant absent mais dans le village, 2=enfant en voyage, 3= Enfant présent mais mère ou tutrice absente, 4=enfant trop malade 5=réaction adverse lors de la précédente administration 6=enfant trop jeune, 7=refus des parents, 9=autre, spécifiez

Si les parents avaient refusé : spécifier la raison du

refus____

<u>Renseignements sur l'Observance des 2ème et 3ème dose de CPS</u>

Lors de la dernière administration de médicaments (Oct/Nov) avez-vous donner la 2ème ou 3ème dose à votre enfant ? 1❑ Oui 2❑ Non

Si non, spécifier la raison pour la 2èmedose : 1❑ oubli 2❑ vomissement 3❑ refus 9❑ Autres spécifier : ……………………………………………… … Lors de la dernière administration de médicaments (Oct/Nov) avez-vous donner la 3ème dose à votre enfant ? 1❑ Oui 2❑ Non

Si non, spécifier la raison pour la 3èmedose : 1❑ oubli 2❑ vomissement 3❑ refus 9❑ Autres spécifier : …………………………………………………

Avez-vous rencontré des difficultés dans l'administration des 2em et 3em doses à domicile ? ? 1❑ Oui 2❑ Non

Si oui, préciser : 1❑ comprimé difficile à administrer, 2❑ oubli de donner le comprimé 3❑refus de l'enfant 4 ❑ vomissement de l'enfant 9❑ Autres, spécifier

Que faites-vous si l'enfant rejette une dose : : 1❑ redonner un comprimé, 2❑ l'amener au CSPS 3❑ l'amener chez l'ASC 9❑ Autres spécifier ………………………..

Que faites-vous si l'enfant vomit une dose : : 1❑ redonner un comprimé, 2❑ amener au CSPS 3❑ l'amener chez l'ASC 9❑ Autres spécifier ………………………..

Votre enfant avait eu-il eu une réaction indésirable suite à la prise de médicaments ? 1❑ Oui 2❑ Non 3❑ Nsp

Si oui, lesquels ? : 1❑ Fièvre/corps chaud 2❑ Diarrhée 3 ❑ Eruption cutanée 4 ❑ Vomissement 5 ❑Perte de l'appétit 6 ❑ Jaunisse 7 ❑ Toux 8 ❑ Prurit 9❑Autres à préciser

Qu'avez- vous fait lors de la survenue d'évènement indésirable ? 1❑ l'amener au CSPS 2❑ l'amener chez l'ASC 3❑ Autre

Etes-vous prêts à accepter que votre enfant prenne la CPS l'année prochaine ? 1❑ Oui 2❑ Non 3❑ Nsp

Si Non, pourquoi ……………………………………
