# Supplementary figures and images for: Factors Influencing Second and Third Dose Observance during Seasonal Malaria Chemoprevention (SMC): A Quantitative Study in Burkina Faso, Mali and Niger
Source: Trop Med Infect Dis. 2022 Aug 29;7(9):214. doi: 10.3390/tropicalmed7090214 (PMC9503675; doi:10.3390/tropicalmed7090214)

# CPS-OOAS: Study area in Burkina Faso

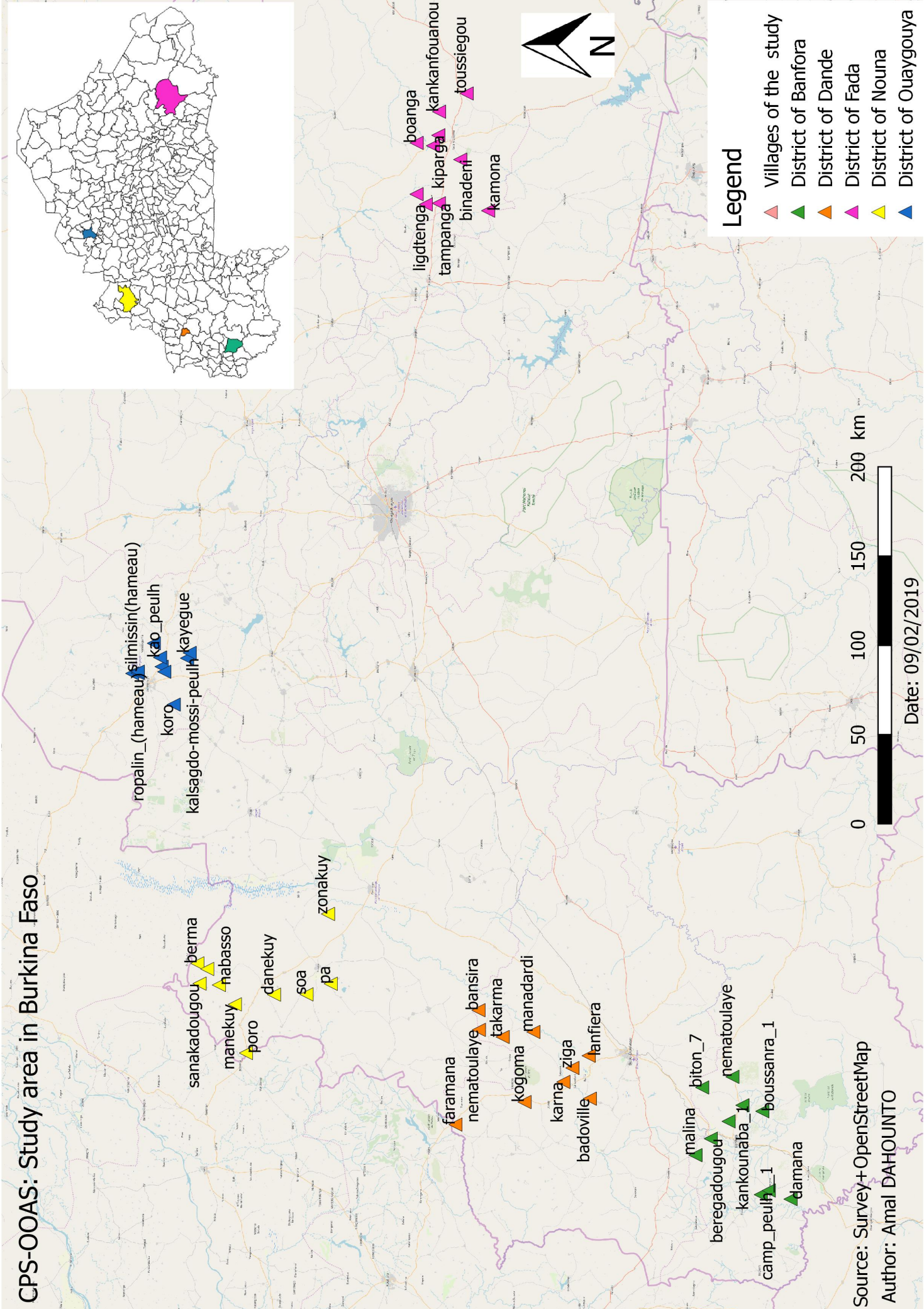

Supplement: Supplementary file 1 [file tropicalmed-07-00214-s001.zip › S1_Geographic representation of study area in Burkina Faso.pdf]

## CPS-OOAS: Study area in Niger

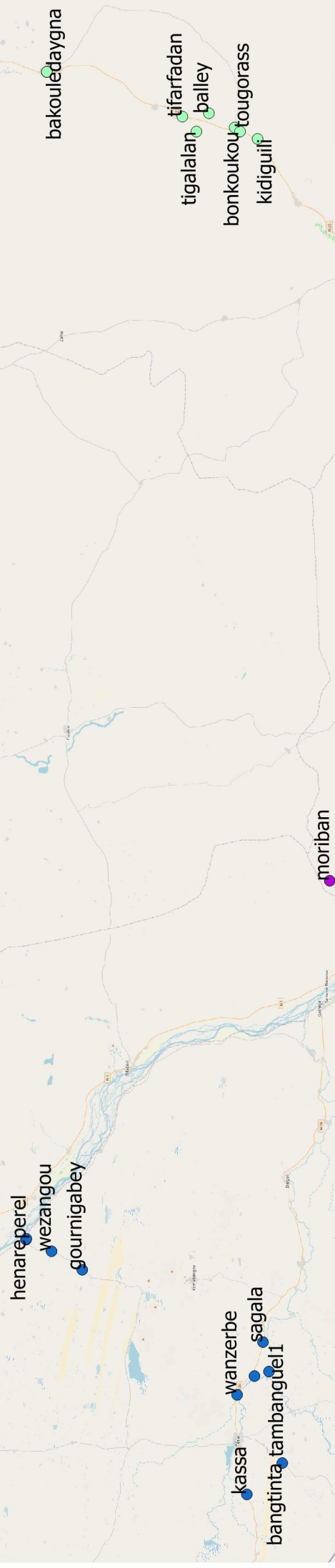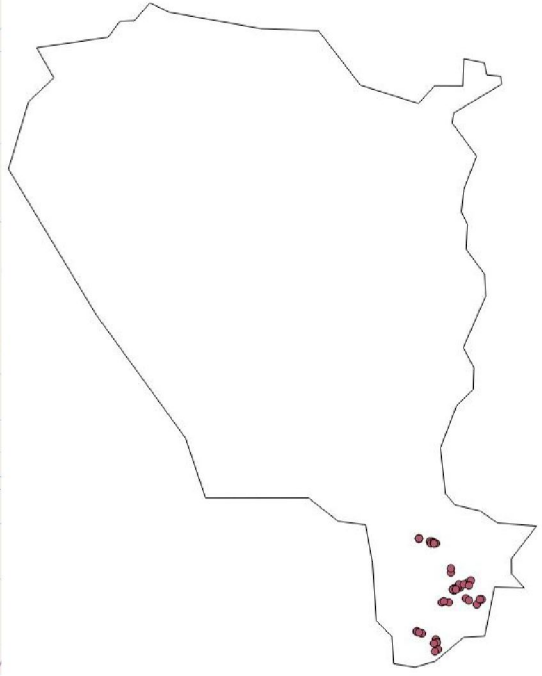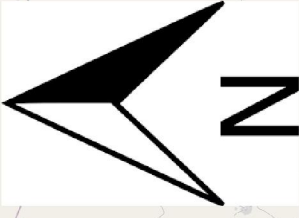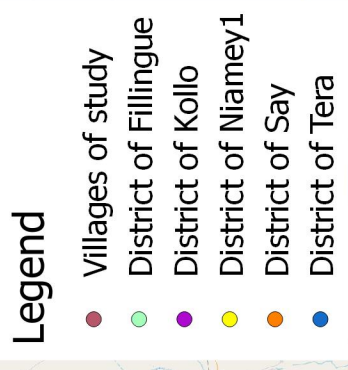

Source: Survey+OpenStreetMap

Author: Amal DAHOUNTO

Date: 2020/08/19

Supplement: Supplementary file 1 [file tropicalmed-07-00214-s001.zip › S3_Geographic representation of study area in Niger.pdf]
